# Systematic review of adherence to technology-based falls prevention programs for community-dwelling older adults: Reimagining future interventions

**DOI:** 10.1371/journal.pdig.0000579

**Published:** 2024-09-03

**Authors:** Maureen C. Ashe, Isis Kelly dos Santos, Jefferson Erome, Jared Grant, Juliana Mollins, Sze-Ee Soh

**Affiliations:** 1 Department of Family Practice, The University of British Columbia (UBC), Vancouver, Canada; 2 Edwin S.H. Leong Centre for Healthy Aging, UBC, Vancouver, Canada; 3 Departament of Physical Education, Federal University of Rio Grande do Norte (UFRN), Natal, Brazil; 4 Department of Physical Therapy, UBC, Vancouver, Canada; 5 Department of Physiotherapy, Monash University, Melbourne, Australia; 6 Rehabilitation, Ageing and Independent Living (RAIL) Research Centre, Monash University, Melbourne, Australia; The University of Hong Kong, HONG KONG

## Abstract

**Background:** Prevention programs, and specifically exercise, can reduce falls among community-dwelling older adults, but low adherence limits the benefits of effective interventions. Technology may overcome some barriers to improve uptake and engagement in prevention programs, although less is known on adherence for providing them via this delivery mode. We aimed to synthesize evidence for adherence to technology-based falls prevention programs in community-dwelling older adults 60 years and older. We conducted a systematic review following standard guidelines to identify randomized controlled trials for remote delivered (i.e., no or limited in-person sessions) technology-based falls prevention programs for community-dwelling older adults. We searched nine sources using Medical Subject Headings (MeSH) terms and keywords (2007-present). The initial search was conducted in June 2023 and updated in December 2023. We also conducted a forward and backward citation search of included studies. Two reviewers independently conducted screening and study assessment; one author extracted data and a second author confirmed findings. We conducted a random effects meta-analysis for adherence, operationalized as participants’ completion of program components, and aimed to conduct meta-regressions to examine factors related to program adherence and the association between adherence and functional mobility. We included 11 studies with 569 intervention participants (average mean age 74.5 years). Studies used a variety of technology, such as apps, exergames, or virtual synchronous classes. Risk of bias was low for eight studies. Five interventions automatically collected data for monitoring and completion of exercise sessions, two studies collected participants’ online attendance, and four studies used self-reported diaries or attendance sheets. Studies included some behavior change techniques or strategies alongside the technology. There was substantial variability in the way adherence data were reported. The mean (range) percent of participants who did not complete planned sessions (i.e., dropped out or lost to follow-up) was 14% (0–32%). The pooled estimate of the proportion of participants who were adherent to a technology-based falls prevention program was 0.82 (95% CI 0.68, 0.93) for studies that reported the mean number of completed exercise sessions. Many studies needed to provide access to the internet, training, and/or resources (e.g., tablets) to support participants to take part in the intervention. We were unable to conduct the meta-regression for adherence and functional mobility due to an insufficient number of studies. There were no serious adverse events for studies reporting this information (n = 8). The use of technology may confer some benefits for program delivery and data collection. But better reporting of adherence data is needed, as well as routine integration and measurement of training and skill development to use technology, and behavior change strategies within interventions. There may be an opportunity to rethink or reimagine how technology can be used to support people’s adoption and integration of physical activity into daily life routines.

## Author summary

Prevention programs, especially exercise-based interventions, can decrease falls risk among older adults living in the community. However, low adherence to programs can impact on the effectiveness of interventions. Using technology may help with some barriers to adopting and continuing with the intervention, although there is limited knowledge of adherence when using this delivery mode for falls prevention programs. We conducted a systematic review of randomized controlled trials for technology-based falls prevention programs for older adults. We were interested in studies that participants completed the interventions at home with minimal in-person contact. After reviewing the included studies, we observed a high level of adherence to interventions but variability among the studies. Other take home messages included the need to better report adherence within programs and provide more information for how programs are implemented. Further, program developers should consider building in time for training and supporting older adults to use technology.

## Introduction

The population is aging globally. By 2050, approximately 22% of the population will be older adults—placing additional pressure on health care systems, the economy, and society [1]. One significant implication of an aging population is the elevated risk for, and consequences of, falls. Annually, one-third of older adults aged over 65 years fall [2], and 30–50% of older adults fall multiple times [2–4]. Falls are the leading cause of older people’s hospitalization [5], and can also result in the development of reduced mobility [6] and disability [7].

Intrinsic changes occur during aging [8], some of which may be modifiable, such as low physical fitness, poor balance, low mobility, increase in anxiety and fear of falling, and medication use [9]. Falls prevention interventions can be single- or multi-component programs designed to prevent and reduce the risk of falls [9]. They may include (but are not limited to) education, risk assessments, footwear advice, medication review, and exercise [10]. Exercise is the most commonly tested single intervention for falls prevention [11]; it is effective [12] and cost-effective [13]. Exercise in general, can also be delivered via different modes (in-person, online, etc.), which may not always be possible or easily completed with other falls prevention interventions, such as a physical exam, home assessment, etc.

To be effective, interventions (in general) need to be adopted (i.e., taken up, started) and completed according to the exercise intervention protocol (adherence) [14]. But there are many reasons (or barriers) why people do not complete an exercise program as planned [15]; the average rate of adherence declines over time [16], and low adherence can limit the effects of exercise prescription [17]. Adherence rates for falls prevention programs vary. Nyman and Victor [18] reported an adherence of 82% for individually targeted exercise programs over a short time frame (10 weeks), which dropped to 52% over 12 months [18]. In-person classes had adherence rates of 83% at two months and 76% at 24 months [18]; while a meta-analysis found only 21% of participants were fully adherent to home-based falls prevention programs [16]. But some differences between studies may be due to how adherence is measured.

Adherence is defined as the *active choice* of participants to follow and complete a prescribed exercise program [19]. In essence, adherence is a measure of people’s behavior—both adoption (uptake or starting exercise) and adherence (or maintenance, creating habits) [20]. Yet, exercise adherence can be confusing because it is frequently reported based on different measures such as completion/retention, attendance (number of sessions completed), duration and/or intensity (exertion) [19,21]. Describing adherence and exploring associated factors are important to integrate into practice, and to increase the chance of benefiting from effective interventions [22].

Information to consider for falls prevention interventions include personal preferences and background (e.g., physical activity history), individualized exercise programs, level and type of supervision, program duration, and number of weekly sessions [23]. Another factor to consider for falls prevention programs using technology may be related to older people’s motivation, digital literacy and self-efficacy, knowledge and skills, support [24,25], and access to resources (e.g., internet, tablets, etc.). In particular, although many older adults use technology regularly [26] and may be interested in learning to use new devices [27], such as tablets [28], this age group may require specific training to adopt a new delivery mode [29], to overcome a possible “digital divide”—defined as “an umbrella term for many issues, including infrastructure and access to ICTs [Information and Communication Technology], use and impediments to use, and the crucial role of ICT literacy and skills to function in an information society.” [30] p. 1. Further, previous work highlights the need to develop technology-based interventions with older adults, that is, co-create programs with them [24,27].

But it is also important to consider behavior change techniques (BCTs) or strategies [31], such as goal setting [23,32] and feedback and monitoring [32], which can support the adoption and maintenance of new behaviors. In general, reviews highlight some BCTs may be effective in supporting older adults to adopt and sustain physical activity [33,34], but it is complicated. Feedback may be an effective strategy to support older adults’ long-term engagement in physical activity [33]. However, another review identified some self-regulatory BCTs (setting goals, feedback), may not always work with older populations [34]. In a more recent systematic review of older adults’ sustainability of physical activity, longer term engagement was associated with adding objects into the environment and feedback (activity trackers), and individualized activity goals [35]. Finally, one systematic review reported the following five BCTs had moderate certainty evidence to encourage program adherence to exercise prescription: social support (unspecified); goal setting (behaviour); instruction of behaviour; demonstration of behaviour; and behaviour practice/rehearsal [36]. While a second systematic review reported goal setting (very low certainty evidence), and self-monitoring and feedback (low certainty evidence) BCTs increased adherence to a physical activity intervention [37].

Technology-based exercise programs may increase people’s motivation and lead to longer-term adherence [32] at relatively lower costs [38]. There has been an increase in remote delivery of exercise (via technology; ICTs), and especially since the start of the COVID-19 pandemic. ICTs are “technological tools and resources used to transmit, store, create, share or exchange information”[39], including computers, the internet, podcasts, and/or video-conferencing. Previous systematic reviews provide preliminary evidence for technology-based exercise programs to improve physical function and reduce falls risk factors in older adults [40–42]. But less is known about the adherence of community-dwelling older adults to engage and continue with technology-delivered falls prevention programs. A 2018 systematic review synthesized evidence comparing technology to traditional exercise programs [38] and noted both delivery modes had high adherence to the program (91% technology-based to 84% traditional programs) [38].

The 2022 World Guidelines for Falls Prevention [9] highlighted a knowledge gap for technology-based falls prevention and management, but they also discussed (for any complex falls prevention intervention), there is a need for behavior change for both people who deliver falls prevention interventions and the people who receive, adopt, and sustain them [9]. Therefore, to address knowledge gaps, we aim to describe adherence to technology-based falls prevention programs in community-dwelling adults aged 60 and older. Given the recent events with the pandemic, we were interested in synthesizing evidence for technology-based interventions with minimal to no in-person contact. Our primary question was: In community-dwelling adults aged 60 and older, what is the adherence to technology-based home falls prevention programs? Our secondary questions were: What factors were associated with program adherence? and What is the association between adherence and functional mobility?

## Methods

This was a systematic review following standard guidelines by the Preferred Reporting Items for Systematic reviews and Meta-Analyses (PRISMA) 2020 statement [43]. This systematic review was registered in PROSPERO (registration number: CRD42023434178). We defined the type of intervention as remote delivered technology-based falls prevention programs; we specifically refer to remote delivery as a program requiring minimal to no in-person contact.

### Eligibility criteria

We used the following criteria to define our search strategy. We only included peer-reviewed randomized controlled trials, and excluded conference abstracts, book chapters, protocols, and grey literature. For ICTs, we included apps, DVDs, exergames, videoconferencing, and videos. We excluded telephone (phone) only based interventions. The decision to not include phone as a delivery mode was because falls prevention programs include exercises and physical activity which may be better delivered via images, apps, or exergames.

*Population*: Community-dwelling adults who were over 60 years of age; or the study’s group mean age was 60 years and older. We included studies with participants with or without a clinical condition, such as mild cognitive impairment or Parkinson’s Disease.

*Intervention*: We focused the search on falls prevention programs (multi-factorial or single factor) delivered remotely (synchronous or asynchronous; and no or limited in-person sessions); although we did permit studies which had some in-person (≤ three) visits to install or demonstrate the intervention. We excluded studies which used a hybrid design of in-person contact with a health provider. This was because we were interested in studies which could be used when in-person care is not possible. We did not limit falls prevention interventions to exercise/physical activity.

*Comparator*: We included studies with any or no comparator (e.g., usual care or wait-list control).

*Outcomes*: For studies to be included, they needed to test a falls prevention intervention (and have any fall-related outcomes) and reported adherence data (e.g., number of sessions or minutes of the intervention).

*Time and Type*: We included only randomized controlled trials, including pilot randomized trials as defined by Eldridge and colleagues [44].

Due to the advances in technology over the past few decades, we limited our search to literature from 2007 and onwards (i.e., from the release of the first iPhone). We included studies from all languages; and decided *a priori* to use an online document translator for publications in languages other than English (e.g., DeepL, Cologne, Germany).

### Information sources and search strategy

We searched the following electronic databases: APA PsycArticles, APA PsycINFO, CINAHL Complete, Cochrane Central Register of Controlled Trials, Embase, Epistemonikos, MEDLINE (Ovid), PEDro, and SPORTDiscus. We also searched Google Scholar using the advanced feature (keywords in title only). Our search strategy included Medical Subject Headings (MeSH) terms and keywords to describe the intervention (remote falls prevention programs delivered via computer, web, video, etc.) and outcomes (measures of adherence at the participant level). One author (MCA) ran all the searches and uploaded citations into Covidence systematic review software, Veritas Health Innovation, Melbourne, Australia (available at www.covidence.org). The search was conducted in June 2023 and updated on December 23, 2023. We also conducted a forward and backward citation search of included studies (December 23, 2023) using Web of Science and Google Scholar.

In **S1 Table.** we provide an overview of our search strategies by databases. We modified and adapted the comprehensive search strategy from Leung and colleagues [45] for Medline, Embase, and Cochrane Central Register of Controlled Trials. We simplified the search for the other databases by using keywords. However, our review differs from the protocol from Leung and colleagues in at least the following ways [45]: we focussed on adherence data (not efficacy/effectiveness data), and we only included interventions which did not include a hybrid approach (in-person and remote delivery).

### Selection process

We followed standard guidelines for screening identified citations: two of three reviewers (MCA, IKS, JG) independently identified citations at Level 1 (abstract and title) and Level 2 (full text) using Covidence software. To be included in the review, studies needed to provide a falls outcome and some adherence data in the publication, however we did email some corresponding authors to clarify specific details (number of sessions/minutes completed).

### Data collection process

We extracted the following information: study type, sample size and study population, participant demographics, program characteristics (e.g., content, method of delivery, duration), method of recording adherence outcomes, and the Timed up and Go (TUG) test [46] or gait speed to examine the association between adherence and functional mobility. We also extracted adverse and serious adverse events using the National Institute of Aging definition of “Any untoward or unfavorable medical occurrence in a human study participant, including any abnormal sign (e.g. abnormal physical exam or laboratory finding), symptom, or disease, temporally associated with the participants’ involvement in the research, whether or not considered related to participation in the research.” [47] p. 1. Based on this same definition, a serious adverse event may include death or risk of death, hospitalization, disability, etc.[47]

For data extraction, one author extracted data (MCA or IKS) and a second or third author confirmed data (MCA, IKS, SES, JM). We also extracted information on relevant BCTs for each study. Based on two systematic reviews on BCTs and adherence to physical activity interventions [36,37] we searched for study information on: demonstration of behavior, behavior practice/rehearsal, goal setting, instruction of behavior, and social support.

### Outcomes: Primary and secondary

Our primary outcome was adherence (operationalized as the number of completed sessions or number of minutes completed) at the participant level. For example, how many classes (sessions) or minutes the participants completed based on the number of prescribed sessions or minutes. We recognise attending classes is not the same as how much (and how intense) people completed the planned intervention [19], but we took a pragmatic approach based on available data. We also did not dichotomize adherence data based on a threshold, such as people completing two-thirds of planned sessions [19]. Our secondary outcome was number of participants who completed the study or final assessment (retention, i.e., did not drop out, or were lost to follow-up). When extracting adherence data, we used available evidence and/or contacted corresponding study authors to confirm data; we aimed to identify number of sessions/minutes planned and number of sessions/minutes completed. When studies reported adherence data at multiple time points, we used data from the follow-up period most comparable with the other included studies, given that adherence to falls prevention programs are likely to decline over time [18].

### Risk of bias assessment

We used the Cochrane Risk of Bias Tool [48] to assess the methodological quality of studies, and a previously published customized checklist to assess sources of bias and ambiguity for adherence data [16]. Two authors independently (MCA, IKS, JM) assessed each study then met to decide the final rating.

### Synthesis methods

We report study characteristics, demographic information, and retention rates using mean (SD), median (IQR) or frequencies. We calculated the proportion of participants adhering to the falls prevention program in two ways: (1) as the number of sessions completed divided by the total planned number of prescribed sessions; and (2) the duration (in minutes) of exercises completed divided by the total expected duration (in minutes) of prescribed exercises. We used standard errors and confidence intervals for a single proportion and transformed them to logits to improve their statistical properties using the following method [49]:

$$logit\;outcome=\ln\left(\frac{p}{1-p}\right)$$

$$logit\;standard\;error=\sqrt{\frac{1}{n\;x\;p\;x(1-p)}}$$

where *p* refers to the proportion of sessions completed, and *n* refers to the total number of expected sessions. To ensure that the confidence intervals of proportions were asymmetrical and did not exceed 0 and 1, variances of the raw proportions were transformed using a Freeman-Tukey arcsine square root transformation. We used the DerSimonian-Laird random effects model to estimate the pooled proportion of adherence to the exercise program to account for between study heterogeneity. We conducted sensitivity analysis examining studies which included only community-dwelling older adults without a reported clinical condition.

In order to determine the association between falls prevention program characteristics and exercise adherence, we used a two-step modelling approach using random effects meta-regression. Univariable models for each program characteristic were examined initially before factors that were significantly associated (*p*≤0.05) with the outcome (i.e., exercise adherence) were simultaneously entered into a multivariable model. We used logit transformed outcomes and within-study standard errors in this set of analyses [50]. We also conducted subgroup analyses based on delivery mode (e.g., exergames, etc.).

We used random effects maximum likelihood meta-regression to examine the association between adherence and functional mobility (operationalized as the TUG or gait speed). The standardised mean differences (SMD) and associated standard error of the estimate were calculated for studies reporting the means (SD) of functional mobility (TUG or gait speed) for both groups. To determine the degree to which program characteristics and adherence explained the variance in trial outcomes, we reported the regression coefficient and associated 95% CI. However, we planned to conduct a meta-regression analysis only if at least 10 trials were included in the meta-analysis, to reduce the risk of generating spurious findings [51]. We used Stata SE 18.0 (StataCorp, LLC) [51] to conduct analyses.

## Results

### Study selection

**Fig 1:**
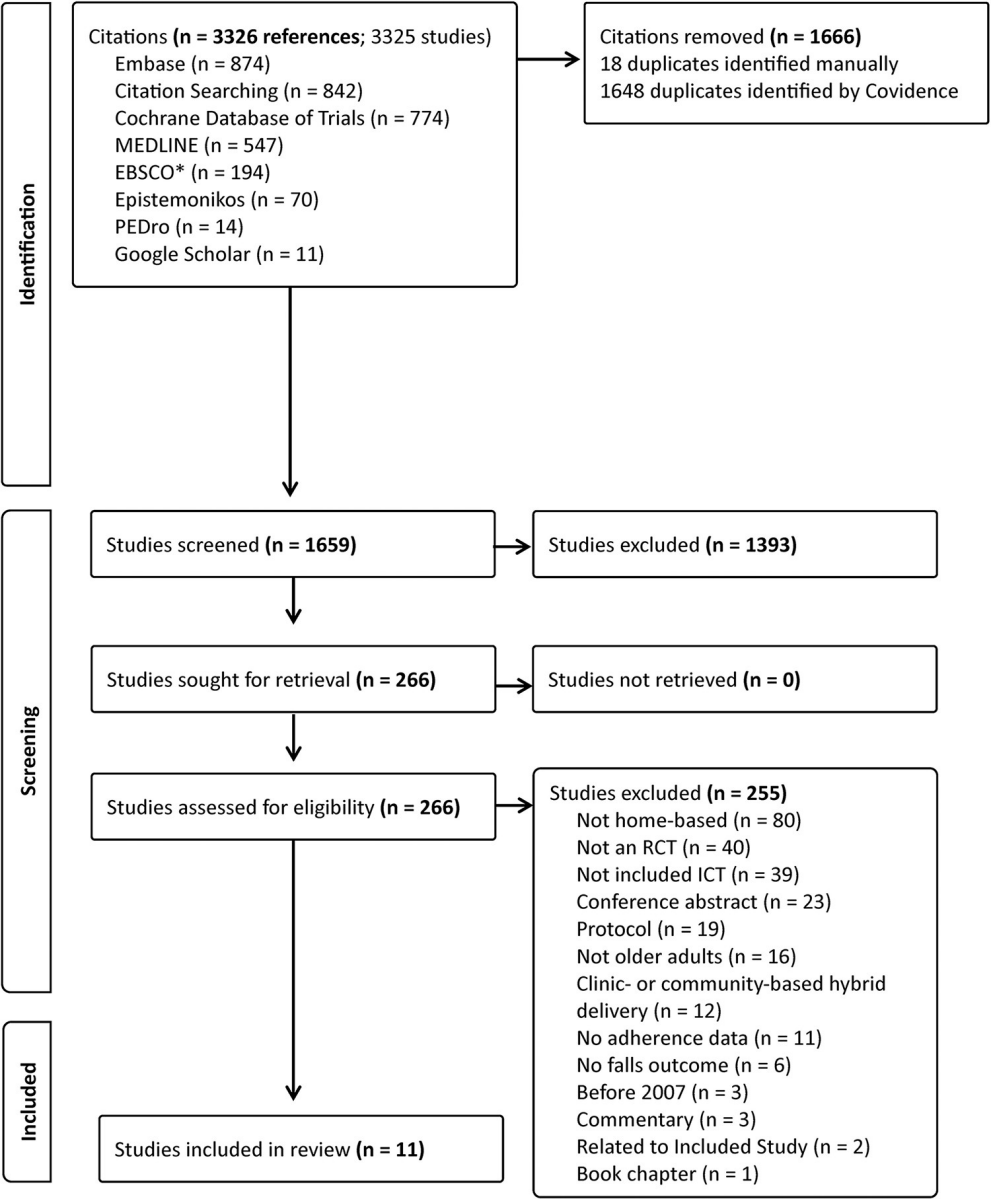
PRISMA flow diagram of the literature search. ICT = Information and communication technology; RCT = randomized controlled trial; *EBSCO searches include the following databases: APA PsycArticles, APA PsycInfo, CINAHL Complete, SPORTDiscus.

We identified 3326 records, and after removal of duplicates 1659 studies were screened by title and abstract and 266 full-text were assessed for eligibility. After full-text review, a total of 11 studies were included in this review [52–62] (**Fig 1 and Table 1**). All studies were published in English. **S2 Table** provides a list of several Level 2 excluded studies and rationale for why they were not included in the systematic review. For the study by Delbaere and colleagues [54], which was the largest and longest duration study (24 months), we used the 6 months adherence values in our analyses, to be comparable with the other included studies, as adherence may decline over time [18].

**Table 1:** Characteristics of included studies (n = 11). We extracted the available data from the included studies and highlight below when it was only available for individual study groups.

Lead Author/year	Country	Study Duration	N	Sample	Age (y) Mean (SD)	Women N (%)	History of falls N (%)
Adcock et al., 2020 [52]	Switzerland	16 weeks	37	Older adults	73.9 (6.4)	16/31 (52%)	5/31 (16%)
Callisaya et al., 2021 [53]	Australia	24 weeks	93	Older adults with CI	72.8 (7.0)	54 (58%)	33 (35%)
Delbaere et al., 2021 [54]	Australia	104 weeks	503	Older adults	77.1 (5.5)*	339 (67%)*	0 (1) median (IQR)
Gschwind et al., 2015 [55]	Australia	16 weeks	153	Older adults	74.7 (6.3)	93 (61%)	51 (33%)
Li et al., 2021 [56]	United States	24 weeks	30	Older adults with MCI	76.1 (6.2)*	21 (70%)	19 (63%)
Schoene et al., 2013 [57]	Australia	8 weeks	37	Older adults in RV	77.5 (4.5)*	NR	9 (24%)
Schoene et al., 2015 [58]	Australia	16 weeks	90	Older adults in RV	81.5 (7)	(66%)*	(38%)*
Song et al., 2018 [59]	Australia	12 weeks	60	Older adults with PD	68 (7)*	36 (60%)*	33 (55%)
Tomita et al., 2016 [60]	United States	24 weeks	56	Older adults	72.3 (7.7)	45 (88%)	100%
Wu et al., 2010 [61]	United States	15 weeks	64	Older adults	76.1 (7.9)*	42 (65%)	16 (25%)*
Yerlikaya, et al, 2021 [62]	Cyprus	8 weeks	18	Older adults with falls risk	70.2 (5.5)*	12 (67%)*	Falls/FOF history

Note: CI = cognitive impairment; FOF = fear of falling; MCI = mild cognitive impairment; IQR = Interquartile Range; NR = not reported; PD = Parkinson’s Disease; RV = retirement village; SD–standard deviation; *Data reported for intervention group only

### Study characteristics

All studies were randomized controlled trials using exercise/physical activity and/or cognition training; and were conducted across four countries: Australia (6 studies) [53–55,57–59], United States (3 studies) [56,60,61], Cyprus (1 study) [62] and Switzerland (1 study) [52]. The average mean age of study participants among studies was 74.5 years. Sample sizes for intervention groups ranged from 15 to 254 community-dwelling older adults. Two studies included community-dwelling older adults with mild cognitive impairment [53,56]; in one study, participants self-reported fear of falling [62]; and one study included older adults with Parkinson’s Disease [59]. Based on 10 studies, on average, 66% percentage of participants were women. The average mean TUG values (for all studies but four [52,53,58,60]) at baseline was 11.65 s (range 8.5–13.4s) and 11.57 (range 8.6–13.4s) for the interventions and control groups, respectively. Gait speed for three studies [52,53,60] at baseline for the intervention group was 1.2 m/s (range 1.07–1.27 m/s) and control group was 1.2 m/s (range 1.1–1.4 m/s). Many studies needed to provide access to the internet, training, and/or resources (e.g., tablets) to support participants to take part in the intervention. Several studies reported developing their technology-based intervention with older adults [52–54].

All falls prevention programs were completed at home using technology to deliver exercise or activities such as: balance training (all studies); muscle strengthening (5 studies) [52,53,55,60,62]; stretching (1 study) [62], Tai Chi (2 studies) [52,61]; and cognitive training (2 studies)[52,53] (**Table 2**). Some studies used an app with videos or platforms to conduct classes [53,54,56,60–62], and five studies used exergames [52,55,57–59]. In eight studies, the amount of recommended duration of exercise per week was 120 minutes or more [52–56,60–62], and all studies had sessions two or more times per week. However, only two studies [55,61] met 150 [9] or 180 [11] minutes/week of physical activity; and all studies but two [57,62] had interventions which lasted 12 weeks or longer. Three studies had no in-person contact [56,61,62], while the following studies had one [52,57,60], two [54,55,58] or three [53,59] in-person home visits to explain/install the programs and for safety reasons. All studies reported phone calls were made to report any falls and/or assist participants with any difficulties with technology. Some studies specifically discussed BCTs embedded in the intervention [53,54], such as adding objects into the environment (a calendar to plan sessions), goal setting [53,54], feedback [52–55,57–60] and providing knowledge (education sheets) [54]. **S3 Table** provides an overview of the BCTs reported for seven BCTs; it is possible studies did use the BCTs, however it was not reported in the publications.

**Table 2:** Characteristics of falls prevention program.

Lead Author/year Location	Falls prevention program	Type of exercise	Delivery mode/ Equipment	Weekly Minutes Frequency	Providers Supervision	Control intervention	In person Contact
Adcock et al., 2020 [52]	Active@Home	Balance, strength and cognitive training	four inertial measurement units TV	120 3 times	Not reported	Usual activities	• Installation and instruction
Callisaya et al., 2021 [53]	Standing Tall program	Balance, strength and cognitive training	App tablet	120 2 times*	EP or PT yes	Monthly phone calls and educational material	• Initial visit for set up • Follow-up visits in weeks 2 and 8 (1h) • Phone calls/Monthly (1h)
Delbaere et al., 2021 [54]	Standing Tall program	Balance	App tablet	120 2 times*	EP no	Educational material and usual care	• Two home visits–intervention group (set-up) • Phone calls–control group
Gschwind et al., 2015 [55]	iStoppFalls	Balance and muscle strength exercises	Exergame Computer, tablet and TV	Up to 180 3 times	Trained research staff no	Educational material	• Two home visits for set-up • Phone calls (if needed)
Li et al., 2021 [56]	Tai Ji Quan	Balance exercises	Zoom Computer, iPad or smartphone	120 2 times	Instructors yes	Stretching group	• Phone calls monthly
Schoene et al., 2013 [57]	Videogame Step Training	Stepping on an electronic pad	Exergame Computer, step pad and TV	Up to 60 2–3 times	NR no	Usual activities	• Initial visit for set-up • Phones calls in weeks 1–3 and 6
Schoene et al., 2015 [58]	Interactive cognitive-motor training	Stepping on an electronic pad	Exergame Computer, step pad and TV	60 3 times	none no	Education and usual activities	• Two visits for set-up • Phones calls in weeks 1, 4, 8 and 12
Song et al., 2018 [59]	Home-based stepping training	Balance	Exergame Computer, step pad and TV	45 3 times	PT no	Usual care	• Two initial home visits (set-up) and at 6 weeks • Phones calls every two weeks
Tomita et al., 2016 [60]	Virtual-Group Exercise at home	Resistance and balance	ooVoo (synchronous online platform) Computer or TV	Up to 120 3 times	EP, FT yes	Walking (in/out doors)	• Initial visit (set-up) • Phone calls monthly
Wu et al., 2010 [61]	Telecommunication-based exercise–Tele-ex group	24-form Yang-style Tai Chi Chuan	DocBox (synchronous online platform) TV	180 3 times	instructor yes	Home–ex group DVD with exercises	• Phones calls every two weeks
Yerlikaya et al, 2021 [62]	Interactive telerehabilitation home exercise group (ITHE)	Resistance and balance	WhatsApp, Google Meet Smartphone and computer	120 3 times	PT yes	None	• Phone calls

EP = exercise physiologist; FT = fitness trainer; PT = physiotherapist;*estimated

### Risk of bias assessment

**Fig 2:**
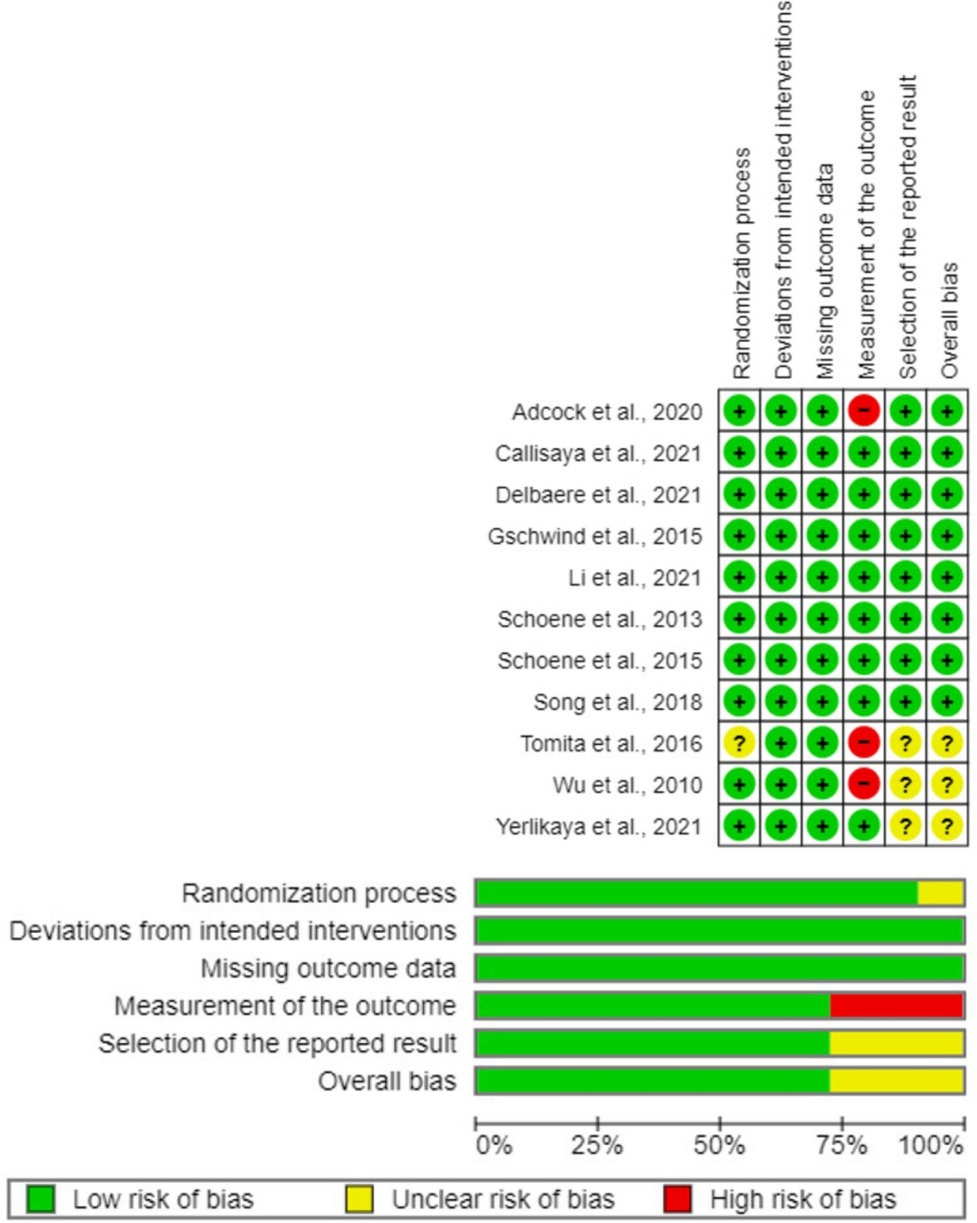
Risk of Bias using Cochrane Risk of Bias tool (RoB2).

Based on our assessment of study risk of bias, eight trials were at low risk of bias overall; and in three trials [60–62] we were unclear about the randomization process, outcome measurement, and/or selection of reported results (**Fig 2**). Information on reporting of adherence data is included in **S4 Table**.

### Adherence to falls prevention programs

Included studies used a variety of methods to measure adherence. Specifically, four studies did not define or operationalize adherence [52,57,59,62]; and there was variability in definitions for studies which provided a definition. Three studies referred to the term “compliance” for adherence [56,57,61]; and three studies discussed participation/attendance [56,60,61]. Five studies reported adherence based on recorded logs, weekly counters and/or graphs generated by apps or exergames [53–55,57,58]. Two studies determined adherence based on the number of sessions attended using videoconferencing [56,60]. Four studies reported data by asking participants to record it on a log sheet provided by the research team [52,59,61,62]. Seven studies reported adherence as the mean (or median) proportion or number of exercise sessions completed [52,57–62]. Two studies reported adherence as a percentage of the minutes prescribed [53,54]. **Table 3** is an overview of each study’s adherence measurement, the percentage of people who completed the intervention, and a description of the reported adverse and serious adverse events. To clarify adherence data (sessions completed) we contacted some corresponding authors.

**Table 3:** Adherence definition and collection, number of people who completed each study, and adverse and serious adverse events.

Study	Adherence			Proportion completed study (%)	Adverse Events	Serious Adverse Events
	Definition	Collection method	Outcome reported			
Adcock et al., 2020 [52]	No definition	Participants’ training diary	• Mean of completed sessions	84	None	None
Callisaya et al., 2021 [53]	Percentage of the minutes prescribed for that week	Via app	• Percentage of participants that adhered to the program	68	Seven adverse events: non-injurious fall using the equipment and six reports of new, or aggravation of existing, musculoskeletal pain.	None
Delbaere et al., 2021 [54]*	Exercise adherence included volume and frequency	Via app after automatic data transfer to a server	• Median minutes exercised per week • Percentage of participants that achieved the prescribed dose	79	Five falls in three participants	None
Gschwind et al., 2015 [55]	High adherence defined as >90 min exercise per week	Monitored via iStoppFalls system	• Median number of times iStoppFalls system used • Median total duration of exercises	81	None	None
Li et al., 2021 [56]	Class participation rate of 75% or better	Attendance collected by research assistant	• Class participation rate • Mean and median number of completed sessions	87	Three participants had non-study related issues	None
Schoene et al., 2013 [57]	No definition	Recordings from computers and self-report	• Median number of sessions played per week • Median duration of exercises completed per session	88	None	None
Schoene et al., 2015 [58]	High adherence based on whether participants played at least 3 games 32 times during the intervention period	Saved by the game computer; monthly calendars (days used and duration of game-play sessions)	• Mean number of sessions played • Mean exercise duration	83	None	None
Song et al., 2018 [59]	No definition	Participants’ paper-based log books	• Mean number of completed sessions	81	Program related: Two participants aggravated existing LBP, and one participant had a fall during training Non-program related: Eight participants had aggravation of pre-existing pain reported as not related to intervention; and one participant fell	Not reported
Tomita et al., 2016 [60]	Attendance in scheduled sessions	Attendance collected by research team	• Percentage of sessions completed with and without make-up sessions	100	Not reported	Not reported
Wu et al., 2010 [61]	Exercise compliance included class attendance, number of minutes exercised in and outside the classes, reasons for missing classes	Participants’ log sheet	• Mean total exercise time • Percentage attendance rate	91	Not reported	Not reported
Yerlikaya et al., 2021 [62]	No definition	Participants’ exercise sheet	• Number of completed sessions	100	Not reported	Not reported

*We used the 6-month results for the study by Delbaere and colleagues.

### Pooled proportion of adherence to technology-based falls prevention programs

**Fig 3:**
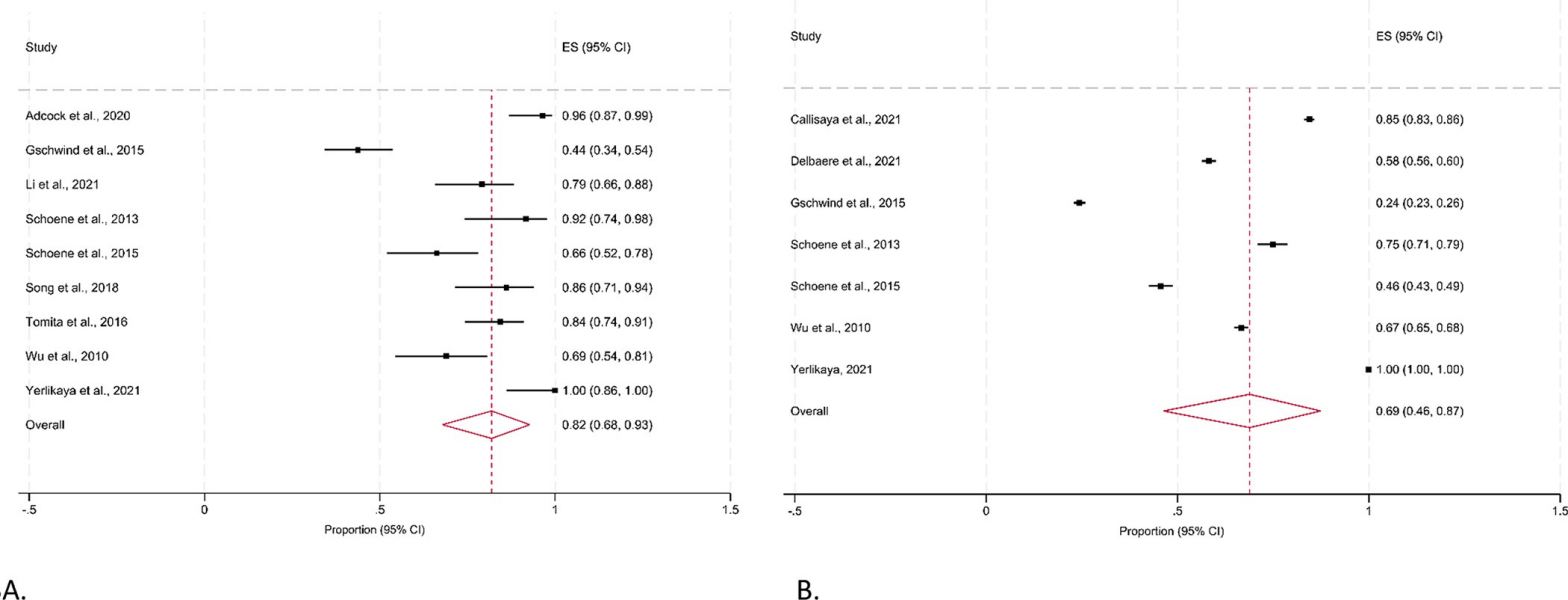
Proportion (95% CI) of community-dwelling older adults adhering to a remote-delivered technology-based falls prevention program in studies reporting the (A) mean number of exercise sessions completed; and (B) duration of exercises completed. **Fig 3A.** Mean number of exercise sessions. **Fig 3B.** Mean number of minutes (duration) of exercise completed.

**Fig 4:**
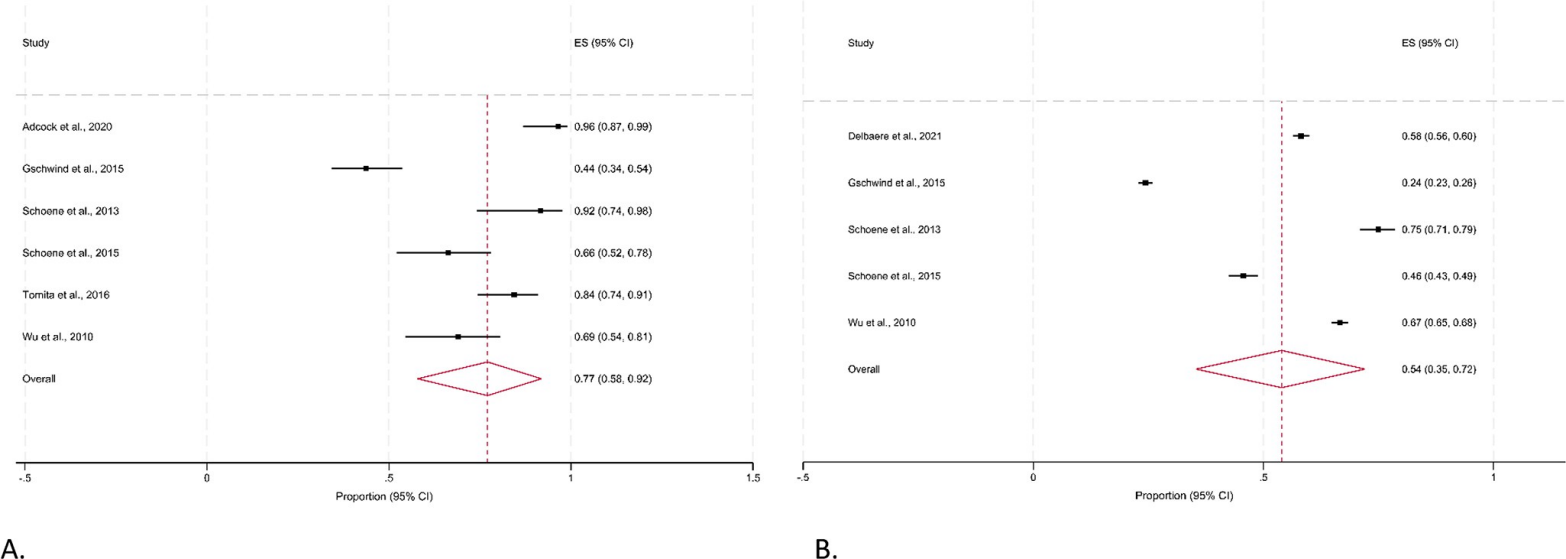
Proportion (95% CI) of community-dwelling older adults without a clinical condition adhering to a remote-delivered falls prevention program in studies reporting the (A) mean number of exercise sessions completed; and (B) duration of exercises completed. **Fig 4A.** Mean number of exercise sessions. **Fig 4B.** Mean number of minutes (duration) of exercise completed.

The pooled estimate of the proportion of participants adherent to programs was 0.82 (95% CI 0.68, 0.93) for studies that reported the mean number of completed exercise sessions. But there was a high level of heterogeneity (I^2^ 91%; Q 89.75; d*f* 8; *p*≤0.001) observed for the adherence rates, reflecting the variability in how adherence was defined and how data were collected (**Fig 3A**). When adherence was estimated using the duration of exercises completed, the pooled proportion of adherence was 0.69 (95% CI 0.46, 0.87). However, higher levels of clinical and methodological heterogeneity were observed as indicated by an I^2^ value of 99% and a Cochrane’s Q figure of 4319.44 (**Fig 3B**). When we only analysed data from studies for older adults without a clinical condition (**Fig 4A and 4B**), we noted a lower pooled adherence rate of 0.77 (95% CI 0.58, 0.92; I^2^ = 93%; p≤0.001) for the number of sessions completed. The pooled proportion of adherence to the duration of exercises completed was also lower in older adults without a clinical condition (0.54, 95% CI 0.35, 0.72), although high levels of heterogeneity was still observed (I^2^ = 99%; Q = 1361.49; df = 4; p≤0.001).

### Factors associated with adherence to technology-based falls prevention program

Given the heterogeneity observed when pooled adherence was estimated using the duration of exercises completed, we included only studies that reported the number of exercise sessions completed. However, none of the characteristics related to falls prevention programs such as program duration, exercise duration or use of follow-up phone calls were associated with exercise adherence in the univariable meta-regression analysis (**Table 4**).

**Table 4:** Univariable meta-regression analysis of the association between technology-based falls prevention program characteristics and exercise adherence.

Program characteristics	Univariable analysis		
	Odds ratio	95% CI	*p*-value
Delivery mode	0.73	(0.08, 6.76)	0.744
Study population	1.19	(0.10, 14.86)	0.869
Mean age	0.92	(0.69, 1.22)	0.485
Baseline functional ability	0.95	(0.37, 2.46)	0.878
Program duration ≥24 weeks	1.12	(0.09, 13.65)	0.913
Exercise duration	0.83	(0.08, 8.09)	0.846
Exercise provider	2.08	(0.18, 23.96)	0.477
Supervision	0.73	(0.08, 6.76)	0.744
Family or friend support	1.37	(0.05, 35.20)	0.820
Follow-up phone calls	1.36	(0.15, 12.60)	0.744
Home visits	1.77	(0.15, 20.35)	0.587
Videoconferencing	0.73	(0.08, 6.76)	0.744
Application-based*	-	-	-
Exergames	1.36	(0.15, 12.60)	0.744

*There were no studies with an app-based intervention which provided the number of exercise sessions completed.

**Fig 5:**
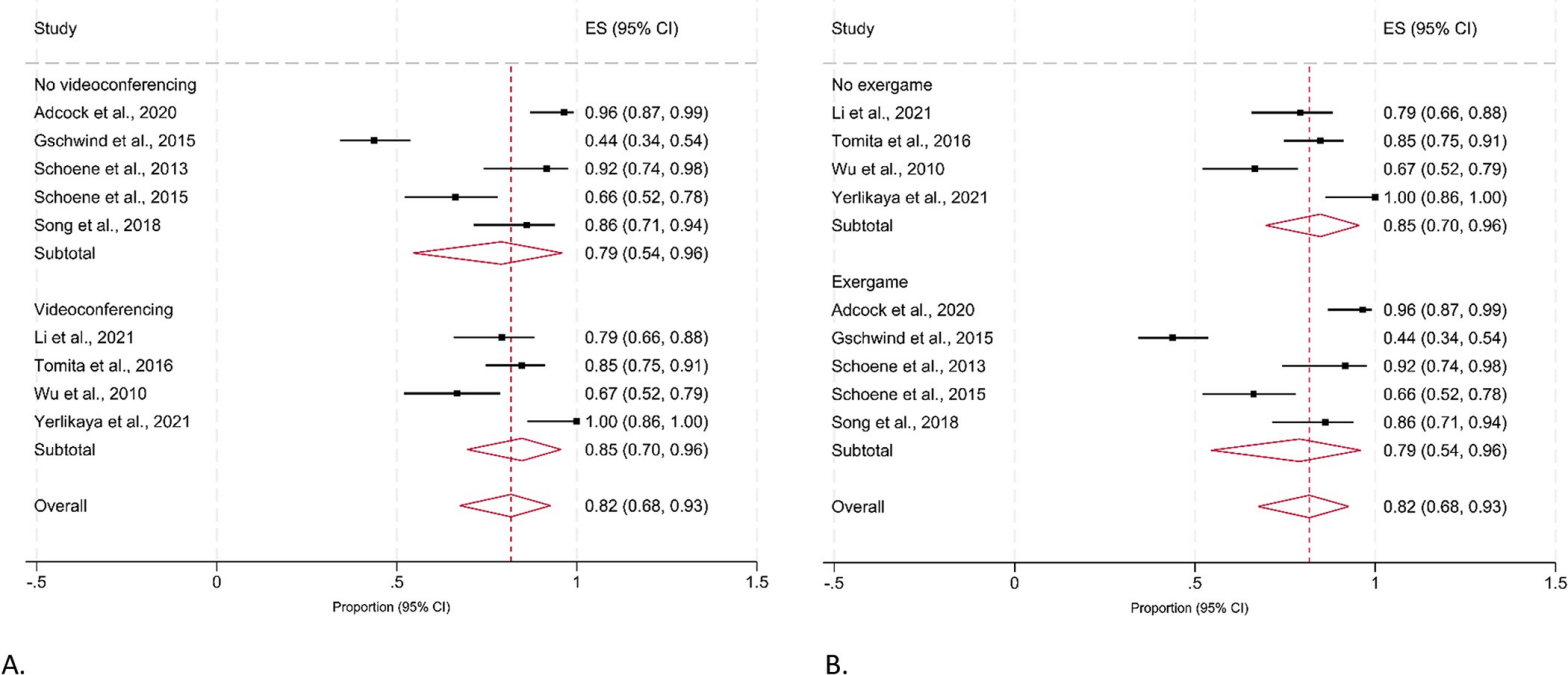
Proportion (95% CI) of participants adhering to falls prevention programs that included (A) videoconferencing; and (B) exergames**. Fig 5A.** Comparison of studies with and without videoconferencing. **Fig 5B.** Comparison of studies with and without exergames.

We conducted subgroup meta-analyses to further explore whether the type of remote delivery contributed to adherence. As shown in **Fig 5A and 5B**, the pooled estimate of adherence rates was slightly higher for programs including videoconferencing (pooled estimate 0.85; 95% CI 0.70, 0.96; I^2^ = 83%) but lower for those that delivered the programs through exergames (pooled estimate 0.79; 95% CI 0.54, 0.96; I^2^ = 94%). We noted considerable heterogeneity across studies (I^2^ = 83% - 94%).

### Association between adherence and functional mobility

Technology-based falls prevention programs appeared to improve functional mobility as measured by the TUG Test or gait speed, but this was not statistically significant (SMD -0.26; 95% CI -0.64, 0.13; I^2^ = 81%; p = 0.187). Given that only nine studies provided sufficient adherence and functional mobility data, this meant that we could not conduct further meta-regression analyses to determine the association between adherence and functional mobility.

## Discussion

In this systematic review we aimed to describe older adults’ adherence to technology-based falls prevention programs. The included studies, generally, had lower risk of bias; and used three different delivery modes: via apps, exergames, and videoconferencing. Some technology was able to automatically collect adherence data; while others relied on self-report data collection. Adverse events were low and there were no serious adverse events for the studies which reported this information. The findings from the meta-analysis highlighted high adherence, although care should be taken when interpreting this result given a high level of observed heterogeneity [63]. This may be a result of variability among study participants. Specifically, not all participant groups were at risk of falling and some participants were living with a chronic condition; and there were differences in the composition of the weekly exercise program (content and weekly duration); and the number of participants who dropped out of the study. The lack of a standardised approach to collecting and reporting adherence data could also have contributed to the observed heterogeneity. Due to the limited number of studies, we were unable to complete the meta-regression for adherence and functional mobility. Finally, few studies specifically mentioned BCTs to support the implementation of the technology-based intervention; although all studies had some form of social support, and many studies provided assistance with setting up the technology and demonstrating the exercise program.

Our findings are consistent with previous literature on adherence to exercise interventions. Nyman and Victor [18] suggested future falls prevention studies (of any type of intervention) could anticipate a “*dropout rate of 10% by 12 months and an 80% adherence rate during the intervention*.” [18] p.21. Although, the studies in this synthesis had a higher mean number of people who dropped out (mean 14%, range 0–32%); and our findings were higher than a previous review of (non-technology-based) home exercise programs, where only 21% of participants adhered fully to the program [16]. We and others also previously noted longer program length was associated with lower adherence to exercise interventions [64,65]. Only one study in this review was longer than 6 months [54], which may also explain the high rate of intervention adherence. Considering the possible differences of programs and participants for the included studies and the high heterogeneity for adherence, there is still promise (in the short term) for technology-based home falls prevention program with limited in-person contact.

Engagement in programs generally declines over time, such that only half of people may still be engaged in falls prevention interventions at 12 months [18]. Longer term maintenance of an intervention needs to be considered to support people to create habits and benefit from exercise or similar health behaviors. It can take a median of 66 days (range 18 to 254 days) to form habits [66]. Therefore, although helpful to understand feasibility of an intervention, shorter term programs may not be long enough for people to embed routines into their daily life. Importantly, automaticity of behavior should be considered as an outcome within trials to discern if and how people incorporate activity into daily lives.

Of note, only two of the included studies specifically mentioned goals [53,54], although the other studies may have used goal setting and pursuit strategies but did not report these specific details. We previously conducted a 4-month group-based in-person feasibility study to incorporate BCTs (including for goal setting and goal pursuit) [67] within an effective falls prevention program [68]. In this single-arm pre-post study, participants reported higher use (compared with baseline) of action control, action planning, habit strength, and exercise self-identity [67]. This program was based on incorporating physical activity (including balance and strength exercises) into everyday routines [68,69], which may be an option for encouraging longer term maintenance of physical activity [35]. The technology-based interventions included in this systematic review could add in more BCTs (if they are not already present), incorporate the information from the programs into daily life routines (e.g., knowledge transfer), and routinely collect and monitor data related to behavior and habit formation.

An advantage of using technology is the ability to add behavior adoption and maintenance strategies within the delivery mode, as was used in several of the included studies. For example, in many of the programs, behavior was monitored automatically when people completed the activities. Further, technology could be used to set goals, and send feedback and reminders. In addition, apps could include a measurement component (self-report and/or change in physical function) which could generate a report for health providers (if permission is given). This may be occurring in practice; however, it was not routinely being reported in the studies for how to deliver falls prevention interventions.

In our review, some studies reported the need to provide access to the internet [53], or tablets/devices to facilitate participation in the falls prevention programs [54,55,57–59,61]. Older adults (in particular) could experience technology barriers, such as difficulty using devices requiring greater support during use; and a lack of technology literacy or access can also result in low self-efficacy, and possible discontinuation of a potentially effective intervention [16,70]. Further, to address the multi-faceted components within the digital divide, there is a need to look at factors beyond age, such as socio-economic status, cultural, and geographic location (environmental) factors, for example [71].

These findings highlight the need to tailor technology-based interventions specifically to older people [38], digital literacy, available resources, and location. It also means more support and training are required for some older people unfamiliar with technology. Implementation of a new delivery mode could take time to help new learners adopt the new technology and new exercise program: Clear instructions are needed to improve older adults’ participation (and reduce the risk for injury) in technology-based interventions delivered without in-person support [54]. Some of these barriers may be overcome by co-creating or developing the intervention and delivery mode with older people [24,27]. Further, as aptly noted in a recent umbrella review, we need to discern what is being adhered to, the technology (e.g., app, exergame) or the exercise [23].

Technology holds promise to support longer term physical activity (exercise) adherence. However, perhaps we need to reimagine interventions delivered via technology rather than trying to replicate in-person classes with a new “tool”. Tapping into the strengths of technology could enhance adoption and maintenance of active living through monitoring and feedback, or other BCTs. However, we observed only one of the included studies [54] specifically mentioned the term BCTs, although several other studies discussed strategies which could positively influence behavior change (for longer term engagement). The use of technology (e.g., adding objects to the environment) is a behavior strategy, but alone, it may not be enough for people to overcome the intention-behavior gap [72].

An important consideration for adherence is social support (another BCT) which can often accompany in-person exercise interventions. Social support can take many other forms, such as via video calls, text messages, or group chats (synchronous or asynchronous). A recent publication commented on the use of artificial intelligence (AI), such as chatbots, for possible use in falls prevention [73], while two reviews synthesized evidence on AI chatbots for lifestyle interventions [74,75]. These are all areas for future research to evaluate as technology-based falls prevention programs evolve. Further, people 60 years and older are a large and diverse group, with different preferences to exercise alone or in a group [76].

Taken together, creating and delivering exercise programs is not “*a one-size-fits all*” and future studies should work with older people to discern the content and delivery mode of falls prevention programs which best supports them. Specifically, studies are needed to develop and test effective strategies to support the uptake and longer-term adherence to technology-based falls prevention programs. In addition, data safety and privacy are of paramount concern for digital and AI-based interventions. It is important to screen people for suitability and preferences for the delivery mode and embed ways to add support and/or supervision. This additional screening and support may encourage program adherence and reduce the risk for adverse events such as falls and pain, due to difficulty with correctly performing exercises [77]. Therefore, new directions in delivering falls prevention programs must include older people within the co-creation process [73].

We note this synthesis has many strengths, such as our decision to only include randomized controlled trials, there was overall low risk of bias for studies’ methods, and processes were completed independently by (at least) two authors and checked by at least one additional author. When data were unclear or not included in the publication, we contacted authors for clarification. Further, we provide a summary of adherence to technology-based falls prevention program delivered with little to no in-person contact, which may be of interest to clinicians and practitioners in the field of aging. However, we also note several systematic review limitations. We only included published studies, and excluded grey literature, therefore publication bias must be considered. Further, the findings are based on a small number of studies, who used a variety of methods to collect and report adherence. This may have contributed to the high statistical heterogeneity observed for pooled adherence rates. Studies only included exercise/physical activity and/or cognition training and did not represent the full spectrum of falls prevention strategies. We did not conduct a full assessment of program BCTs, as this was not a main aim of the review. However, we did provide an overview of some BCTs found to have some evidence in previous studies.

## Conclusions

This synthesis suggests falls prevention programs delivered via technology with minimal to no in-person contact have high adherence but high variability, but these are findings are from a limited number of small studies. Despite the promise of technology to deliver and record people’s uptake and adherence to falls prevention programs, the field would benefit from better and more consistent reporting. Further, future studies should continue to co-create new falls prevention exercise programs with older people and providers and develop and test effective strategies to support program adoption and maintenance while re-imagining how best to safely incorporate more of the advantages provided by technology.

## Supporting information

S1 Table List of database searches. The initial searches were complete in June 2023 and updated on December 23, 2023.
(PDF)

S2 Table List of selected excluded studies with rationale. (PDF)

S3 Table Behavior change techniques (BCTs) reported in the studies (listed by first author and year) following the taxonomy by Michie and Colleagues [31]. We only searched studies for BCTs which were previously identified with adherence to physical activity interventions [36,37]. If the BCT was reported, we scored it as “1 = yes”, or “0 = no”. If information was not provided, we used “NR”.
(PDF)

S4 Table Assessment of risk of bias for reporting adherence data. (PDF)
