# Supplementary material for: Systematic review of adherence to technology-based falls prevention programs for community-dwelling older adults: Reimagining future interventions
Source: PLOS Digit Health. 2024 Sep 3;3(9):e0000579. doi: 10.1371/journal.pdig.0000579 (PMC11371225; doi:10.1371/journal.pdig.0000579)
Supplement: S1 Table — The initial searches were complete in June 2023 and updated on December 23, 2023. (PDF) [file pdig.0000579.s001.pdf]

S1 Table: List of database searches. The initial searches were complete in June 2023 and updated on December 23, 2023.

Database or Search Engine	Search Strategy
Medline, Embase, Cochrane Central Register of Controlled Trials<br><br>Ovid MEDLINE(R) and Epub Ahead of Print, In-Process, In-Data-Review & Other Non-Indexed Citations, Daily and Versions 1946 to December 23, 2023<br><br>Embase 1974 to 2023 December 22, 2023<br><br>EBM Reviews - Cochrane Central Register of Controlled Trials November 2023	The searches for Medline, Embase, Cochrane Central Register of Controlled Trials were modified and adapted from the strategy developed by Leung and colleagues [1].
EBSCO APA PsycArticles, APA PsycInfo, CINAHL Complete, SPORTDiscus<br><br>December 23, 2023	(falls reduction or fall prevention or decreased falls or reduce falls or falls prevention) AND ("in home" or "home based" or remote delivery or telehealth or tele-health or telemedicine or tele-medicine or telerehabilitation or tele-rehabilitation or exergam* or virtual reality) AND (randomised controlled trial or randomized controlled trial or RCT) NOT TI (review of literature or literature review or meta-analysis or systematic review) NOT TI protocol
Epistemonikos<br>Primary study<br><br>December 23, 2023	(fall prevention OR falls prevention) AND (remote delivery OR telehealth OR tele-health OR telemedicine OR tele-medicine OR telerehabilitation OR tele-rehabilitation)
Google Scholar<br><br>December 23, 2023	allintitle: "falls prevention" OR "fall prevention" telehealth OR "tele health" OR telemedicine OR "tele medicine" OR telerehabilitation OR "tele rehabilitation" -protocol -review
PEDro (Physiotherapy Evidence Database)<br>Gerontology<br>Clinical Trial<br>December 23, 2023	Searched for “falls prevention” AND (separately) app OR web-based OR online OR telehealth OR telerehabilitation OR mhealth OR eHealth OR remote OR exergames

1. Leung KK, Carr FM, Kennedy M, Russell MJ, Sari Z, Triscott JA, et al. Effectiveness of telerehabilitation and home-based falls prevention programs for community-dwelling older adults: a systematic review and meta-analysis protocol. BMJ Open. 2023;13(4):e069543. Epub 20230421..
