# Supplementary material for: Systematic review of adherence to technology-based falls prevention programs for community-dwelling older adults: Reimagining future interventions
Source: PLOS Digit Health. 2024 Sep 3;3(9):e0000579. doi: 10.1371/journal.pdig.0000579 (PMC11371225; doi:10.1371/journal.pdig.0000579)
Supplement: S2 Table — (PDF) [file pdig.0000579.s002.pdf]

S2 Table. List of selected excluded studies with rationale.

First Author, Year	Reason for the exclusion in the systematic review
Bernocchi et al., 2019 [1]	Not an included technology
Gandolfi et al., 2017 [2]	No adherence data
Grau-Pellicer et al., 2020 [3]	Not home-based
Goffredo et al., 2023 [4]	No adherence data
Gustafson et al., 2022 [5]	No adherence data
Gschwind et al.,2015b [6]	Not an RCT
Hawley-Hague et al., 2023 [7]	Regular in-person visits
Hong et al., 2018 [8]	No adherence data
Khalil et al., 2019 [9]	Not older adults
Lee et al., 2022 [10]	Not home-based
Lee et al., 2023 [11]	No adherence data
Lim et al., 2023 [12]	No adherence data
Oba et al., 2022 [13]	No adherence data
Spink et al., 2012 [14]	Not an included technology
Thaut et al., 2019 [15]	No adherence data
Van Ravenstein 2020 [16]	Not an RCT
Yi and Yim, 2021 [17]	No adherence data

References

1. Bernocchi P, Giordano A, Pintavalle G, Galli T, Ballini Spoglia E, Baratti D, et al. Feasibility and Clinical Efficacy of a Multidisciplinary Home-Telehealth Program to Prevent Falls in Older Adults: A Randomized Controlled Trial. J Am Med Dir Assoc. 2019;20(3):340-6. Epub 20181023. doi: 10.1016/j.jamda.2018.09.003. PubMed PMID: 30366759.

2. Gandolfi M, Geroin C, Dimitrova E, Boldrini P, Waldner A, Bonadiman S, et al. Virtual Reality Telerehabilitation for Postural Instability in Parkinson's Disease: A Multicenter, Single-Blind, Randomized, Controlled Trial. Biomed Res Int. 2017;2017((Gandolfi, Geroin, Dimitrova, Picelli, Vale, Smania) Neuromotor and Cognitive Rehabilitation Research Center (CRRNC), Department of Neurosciences, Biomedicine and Movement Sciences, University of Verona, P.le L.A. Scuro 10, Verona 37134, Italy(Gandolfi, P):7962826. Epub 20171126. doi: 10.1155/2017/7962826. PubMed PMID: 29333454; PubMed Central PMCID: PMCPMC5733154.

3. Grau-Pellicer M, Lalanza JF, Jovell-Fernandez E, Capdevila L. Impact of mHealth technology on adherence to healthy PA after stroke: a randomized study. Top Stroke Rehabil. 2020;27(5):354-68. Epub 20191202. doi: 10.1080/10749357.2019.1691816. PubMed PMID: 31790639.

4. Goffredo M, Pagliari C, Turolla A, Tassorelli C, Di Tella S, Federico S, et al. Non-Immersive Virtual Reality Telerehabilitation System Improves Postural Balance in People with Chronic Neurological Diseases. J Clin Med. 2023;12(9):3178. Epub 20230428. doi: 10.3390/jcm12093178. PubMed PMID: 37176618; PubMed Central PMCID: PMCPMC10179507.

5. Gustafson DH, Sr., Kornfield R, Mares ML, Johnston DC, Cody OJ, Yang EF, et al. Effect of an eHealth intervention on older adults' quality of life and health-related outcomes: a randomized clinical trial. J Gen Intern Med. 2022;37(3):521-30. Epub 20210607. doi: 10.1007/s11606-021-06888-1. PubMed PMID: 34100234; PubMed Central PMCID: PMCPMC8183591.

6. Gschwind YJ, Schoene D, Lord SR, Ejupi A, Valenzuela T, Aal K, et al. The effect of sensor-based exercise at home on functional performance associated with fall risk in older people–a comparison of two exergame interventions. Eur Rev Aging Phys Act. 2015;12(1):1-9.

7. Hawley-Hague H, Tacconi C, Mellone S, Martinez E, Yang F, Su TL, et al. Using Smartphone TechnolOGy to Support an EffecTive Home ExeRcise Intervention to Prevent Falls amongst Community-Dwelling Older Adults: The TOGETHER Feasibility RCT. Gerontology. 2023;69(6):783-98. Epub 20221205. doi: 10.1159/000528471. PubMed PMID: 36470216; PubMed Central PMCID: PMCPMC10273876.

8. Hong J, Kong HJ, Yoon HJ. Web-Based Telepresence Exercise Program for Community-Dwelling Elderly Women With a High Risk of Falling: Randomized Controlled Trial. JMIR Mhealth Uhealth. 2018;6(5):e132. Epub 20180528. doi: 10.2196/mhealth.9563. PubMed PMID: 29807877; PubMed Central PMCID: PMCPMC5996181.

9. Khalil H, Al-Sharman A, El-Salem K, Alghwiri AA, Al-Shorafat D, Khazaaleh S, et al. The development and pilot evaluation of virtual reality balance scenarios in people with multiple sclerosis (MS): A feasibility study. NeuroRehabilitation. 2018;43(4):473-82. doi: 10.3233/NRE-182471. PubMed PMID: 30400117.

10. Lee EL, Ko MH, Shin MJ, Lee BJ, Jung DH, Han KS, et al. The Effect of Convergence Gamification Training in Community-Dwelling Older People: A Multicenter, Randomized Controlled Trial. J Am Med Dir Assoc. 2022;23(3):373-8 e3. Epub 20210630. doi: 10.1016/j.jamda.2021.05.041. PubMed PMID: 34216552.

11. Lee JI, Park J, Koo J, Son M, Hwang JH, Lee JY, et al. Effects of the home-based exercise program with an augmented reality system on balance in patients with stroke: a randomized controlled trial. Disabil Rehabil. 2023;45(10):1705-12. Epub 20220515. doi: 10.1080/09638288.2022.2074154. PubMed PMID: 35574910.

12. Lim ML, Tran M, van Schooten KS, Radford KA, O'Dea B, Baldwin P, et al. A Self-Guided Online Cognitive Behavioural Therapy to Reduce Fear of Falling in Older People: a Randomised Controlled Trial. Int J Behav Med. 2023;30(3):455-62. Epub 20220602. doi: 10.1007/s12529-022-10105-6. PubMed PMID: 35655058.

13. Oba K, Kagiwada Y, Kamada M, Miki R, Kondo Y, Kamakura T, et al. Evaluating the feasibility of a remote-based training program supported by information and communications technology in the older adults living at home. BMC Geriatr. 2022;22(1):574. Epub 20220713. doi: 10.1186/s12877-022-03273-3. PubMed PMID: 35831789; PubMed Central PMCID: PMCPMC9277784.

14. Spink MJ, Fotoohabadi MR, Wee E, Landorf KB, Hill KD, Lord SR, et al. Predictors of adherence to a multifaceted podiatry intervention for the prevention of falls in older people. BMC Geriatr. 2011;11(1):51. Epub 20110826. doi: 10.1186/1471-2318-11-51. PubMed PMID: 21871080; PubMed Central PMCID: PMCPMC3224214.

15. Thaut MH, Rice RR, Braun Janzen T, Hurt-Thaut CP, McIntosh GC. Rhythmic auditory stimulation for reduction of falls in Parkinson's disease: a randomized controlled study. Clin Rehabil. 2019;33(1):34-43. Epub 20180723. doi: 10.1177/0269215518788615. PubMed PMID: 30033755.

16. VanRavenstein K, Brotherton S, Davis B. Investigating the Feasibility of Using Telemedicine to Deliver a Fall Prevention Program: A Pilot Study. J Allied Health. 2020;49(3):221-7. PubMed PMID: 32877481.

17. Yi D, Yim J. Remote Home-Based Exercise Program to Improve the Mental State, Balance, and Physical Function and Prevent Falls in Adults Aged 65 Years and Older During the COVID-19 Pandemic in Seoul, Korea. Med Sci Monit. 2021;27:e935496. Epub 20211221. doi: 10.12659/MSM.935496. PubMed PMID: 34930888; PubMed Central PMCID: PMCPMC8710034.
