# Supplementary material for: Systematic review of adherence to technology-based falls prevention programs for community-dwelling older adults: Reimagining future interventions
Source: PLOS Digit Health. 2024 Sep 3;3(9):e0000579. doi: 10.1371/journal.pdig.0000579 (PMC11371225; doi:10.1371/journal.pdig.0000579)
Supplement: S3 Table — We only searched studies for BCTs which were previously identified with adherence to physical activity interventions [36,37]. If the BCT was reported, we scored it as “1 = yes”, or “0 = no”. If information was not provided, we used “NR”. (PDF) [file pdig.0000579.s003.pdf]

S3 Table. Behavior change techniques (BCTs) reported in the studies (listed by first author and year) following the taxonomy by Michie and Colleagues [1]. We only searched studies for BCTs which were previously identified with adherence to physical activity interventions [2]. If the BCT was reported, we scored it as “1=yes”, or “0=no”. If information was not provided, we used “NR”.

Behavior Change Technique (BCT)	Adcock 2020 [3]	Callisaya 2021 [4]	Delbaere 2021 [5]	Gschwind 2015 [6]	Li 2021 [7]	Schoene 2013 [8]	Schoene 2015 [9]	Song 2018 [10]	Tomita 2016 [11]	Wu 2010 [12]	Yerlikaya, 2021 [13]
Behavior practice (rehearsal)	1	1	1	1	1	1	1	1	1	1	1
Demonstration of behaviour	1	1	1	1	1	1	1	1	1	1	1
Feedback	1	1	1	1	NR	1	1	1	1	1	1
Goal setting	NR	1	1	NR	NR	NR	NR	NR	NR	NR	NR
Instruction of behaviour	1	1	1	1	1	1	1	1	1	1	1
Self-monitoring	1	1	1	1	NR	1	1	1	NR	NR	NR
Social support	1	1	1	1	1	1	1	1	1	1	1

References

1. Michie S, Richardson M, Johnston M, Abraham C, Francis J, Hardeman W, et al. The behavior change technique taxonomy (v1) of 93 hierarchically clustered techniques: building an international consensus for the reporting of behavior change interventions. Ann Behav Med. 2013;46(1):81-95. Epub 2013/03/21. doi: 10.1007/s12160-013-9486-6. PubMed PMID: 23512568.

2. Meade LB, Bearne LM, Sweeney LH, Alageel SH, Godfrey EL. Behaviour change techniques associated with adherence to prescribed exercise in patients with persistent musculoskeletal pain: Systematic review. Br J Health Psychol. 2019;24(1):10-30. Epub 20180617. doi: 10.1111/bjhp.12324. PubMed PMID: 29911311; PubMed Central PMCID: PMCPMC6585717; Peiris CL, Gallagher A, Taylor NF, McLean S. Behavior Change Techniques Improve Adherence to Physical Activity Recommendations for Adults with Metabolic Syndrome: A Systematic Review. Patient Prefer Adherence. 2023;17(null):689-97. Epub 20230315. doi: 10.2147/PPA.S393174. PubMed PMID: 36945683; PubMed Central PMCID: PMCPMC10024875.

3. Adcock M, Fankhauser M, Post J, Lutz K, Zizlsperger L, Luft AR, Guimarães V, Schättin A and de Bruin ED. Effects of an In-home Multicomponent Exergame Training on Physical Functions, Cognition, and Brain Volume of Older Adults: A Randomized Controlled Trial. Front. Med. 2020; 6:321. doi: 10.3389/fmed.2019.00321

4. Callisaya ML, Jayakody O, Vaidya A, Srikanth V, Farrow M, Delbaere K. A novel cognitive-motor exercise program delivered via a tablet to improve mobility in older people with cognitive impairment–StandingTall Cognition and Mobility. Exp Gerontol. 2021;152:111434.

5. Delbaere K, Valenzuela T, Lord SR, Clemson L, Zijlstra GAR, Close JCT, et al. E-health StandingTall balance exercise for fall prevention in older people: results of a two year randomised controlled trial. BMJ. 2021;373:n740. Epub 20210406. doi: 10.1136/bmj.n740. PubMed PMID: 33824131; PubMed Central PMCID: PMCPMC8022322.

6. Gschwind YJ, Eichberg S, Ejupi A, de Rosario H, Kroll M, Marston HR, et al. ICT-based system to predict and prevent falls (iStoppFalls): results from an international multicenter randomized controlled trial. Eur Rev Aging Phys Act. 2015;12:10. Epub 20151127. doi: 10.1186/s11556-015-0155-6. PubMed PMID: 26865874; PubMed Central PMCID: PMCPMC4748323.

7. Li F, Harmer P, Voit J, Chou LS. Implementing an Online Virtual Falls Prevention Intervention During a Public Health Pandemic for Older Adults with Mild Cognitive Impairment: A Feasibility Trial. Clin Interv Aging. 2021;16(101273480):973-83. Epub 20210525. doi: 10.2147/CIA.S306431. PubMed PMID: 34079243; PubMed Central PMCID: PMCPMC8164667.

8. Schoene D, Lord SR, Delbaere K, Severino C, Davies TA, Smith ST. A randomized controlled pilot study of home-based step training in older people using videogame technology. PLoS One. 2013;8(3):e57734. Epub 20130305. doi: 10.1371/journal.pone.0057734. PubMed PMID: 23472104; PubMed Central PMCID: PMCPMC3589451.

9. Schoene D, Valenzuela T, Toson B, Delbaere K, Severino C, Garcia J, et al. Interactive Cognitive-Motor Step Training Improves Cognitive Risk Factors of Falling in Older Adults - A Randomized Controlled Trial. PLoS One. 2015;10(12):e0145161. Epub 20151216. doi: 10.1371/journal.pone.0145161. PubMed PMID: 26673919; PubMed Central PMCID: PMCPMC4682965.

10. Song J, Paul SS, Caetano MJD, Smith S, Dibble LE, Love R, et al. Home-based step training using videogame technology in people with Parkinson's disease: a single-blinded randomised controlled trial. Clin Rehabil. 2018;32(3):299-311. Epub 20170726. doi: 10.1177/0269215517721593. PubMed PMID: 28745063.

11. Tomita M, Fisher N, Ramsey D, Stanton K, Bierdeman L, Kocher L, et al. Effects of virtual-group exercise at home (V-GEAH) on adherence and fall risks in older adults with a history of falling. Gerontology & Geriatrics Research. 2016;2(3):1018.

12. Wu G, Keyes L, Callas P, Ren X, Bookchin B. Comparison of telecommunication, community, and home-based Tai Chi exercise programs on compliance and effectiveness in elders at risk for falls. Arch Phys Med Rehabil. 2010;91(6):849-56. doi: 10.1016/j.apmr.2010.01.024. PubMed PMID: 20510973.

13. Yerlikaya T, Öniz A, Özgùren M. The effect of an interactive tele rehabilitation program on balance in older individuals. Neurological Sciences and Neurophysiology. 2021;38(3):180-6.
