# Supplementary material for: Systematic review of adherence to technology-based falls prevention programs for community-dwelling older adults: Reimagining future interventions
Source: PLOS Digit Health. 2024 Sep 3;3(9):e0000579. doi: 10.1371/journal.pdig.0000579 (PMC11371225; doi:10.1371/journal.pdig.0000579)
Supplement: S4 Table — (PDF) [file pdig.0000579.s004.pdf]

**S4 Table.** Assessment of risk of bias for reporting adherence data.

| Author/year | Participants and design | Adherence recording | Timing of collection | Period of data recall | Description of training | Data verification | Missing data | Total |
|---|---|---|---|---|---|---|---|---|
| Adcock et al., 2020 [1] | 1 | 1 | 1 | 0 | 1 | 0 | 0 | **4** |
| Callisaya et al., 2021 [2] | 1 | 1 | 1 | Unclear | 1 | 1 | 1 | **6** |
| Delbaere et al., 2021 [3] | 1 | Unclear | 1 | 1 | 1 | Unclear | 0 | **4** |
| Gschwind et al., 2015 [4] | 1 | 1 | Unclear | 0 | 1 | Unclear | 0 | **3** |
| Li et al., 2021 [5] | 1 | 0 | 0 | 0 | 1 | 1 | 0 | **3** |
| Schoene et al., 2013 [6] | 1 | 1 | 0 | 0 | 1 | 0 | 0 | **3** |
| Schoene et al., 2015 [7] | 1 | 1 | 0 | 0 | 1 | 0 | 0 | **3** |
| Song et al., 2018 [8] | 1 | 0 | 0 | 0 | 1 | 0 | 0 | **2** |
| Tomita et al., 2016 [9] | 1 | 1 | 0 | 0 | 1 | 0 | 1 | **4** |
| Wu et al., 2010 [10] | 1 | 1 | 1 | 0 | 1 | 1 | 1 | **6** |
| Yerlikaya et al., 2021 [11] | 1 | 0 | 0 | 0 | 1 | 0 | 0 | **2** |
| **Total** | **11/11** | **7/11** | **4/11** | **1/11** | **11/11** | **3/11** | **3/11** | |

**References**


1. Adcock M, Fankhauser M, Post J, Lutz K, Zizlsperger L, Luft AR, Guimarães V, Schättin A and de Bruin ED (2020) Effects of an In-home Multicomponent Exergame Training on Physical Functions, Cognition, and Brain Volume of Older Adults: A Randomized Controlled Trial. Front Med 2020;6:321. doi: 10.3389/fmed.2019.00321.

2. Callisaya ML, Jayakody O, Vaidya A, Srikanth V, Farrow M, Delbaere K. A novel cognitive-motor exercise program delivered via a tablet to improve mobility in older people with cognitive impairment–StandingTall Cognition and Mobility. Exp Gerontol. 2021;152:111434.

3. Delbaere K, Valenzuela T, Lord SR, Clemson L, Zijlstra GAR, Close JCT, et al. E-health StandingTall balance exercise for fall prevention in older people: results of a two year randomised controlled trial. BMJ. 2021;373:n740. Epub 20210406. doi: 10.1136/bmj.n740. PubMed PMID: 33824131; PubMed Central PMCID: PMCPMC8022322.

4. Gschwind YJ, Eichberg S, Ejupi A, de Rosario H, Kroll M, Marston HR, et al. ICT-based system to predict and prevent falls (iStoppFalls): results from an international multicenter randomized controlled trial. Eur Rev Aging Phys Act. 2015;12:10. Epub 20151127. doi: 10.1186/s11556-015-0155-6. PubMed PMID: 26865874; PubMed Central PMCID: PMCPMC4748323.

5. Li F, Harmer P, Voit J, Chou LS. Implementing an Online Virtual Falls Prevention Intervention During a Public Health Pandemic for Older Adults with Mild Cognitive Impairment: A Feasibility Trial. Clin Interv Aging. 2021;16(101273480):973-83. Epub 20210525. doi: 10.2147/CIA.S306431. PubMed PMID: 34079243; PubMed Central PMCID: PMCPMC8164667.

6. Schoene D, Lord SR, Delbaere K, Severino C, Davies TA, Smith ST. A randomized controlled pilot study of home-based step training in older people using videogame technology. PLoS One. 2013;8(3):e57734. Epub 20130305. doi: 10.1371/journal.pone.0057734. PubMed PMID: 23472104; PubMed Central PMCID: PMCPMC3589451.

7. Schoene D, Valenzuela T, Toson B, Delbaere K, Severino C, Garcia J, et al. Interactive Cognitive-Motor Step Training Improves Cognitive Risk Factors of Falling in Older Adults - A Randomized Controlled Trial. PLoS One. 2015;10(12):e0145161. Epub 20151216. doi: 10.1371/journal.pone.0145161. PubMed PMID: 26673919; PubMed Central PMCID: PMCPMC4682965.

8. Song J, Paul SS, Caetano MJD, Smith S, Dibble LE, Love R, et al. Home-based step training using videogame technology in people with Parkinson's disease: a single-blinded randomised controlled trial. Clin Rehabil. 2018;32(3):299-311. Epub 20170726. doi: 10.1177/0269215517721593. PubMed PMID: 28745063.

9. Tomita M, Fisher N, Ramsey D, Stanton K, Bierdeman L, Kocher L, et al. Effects of virtual-group exercise at home (V-GEAH) on adherence and fall risks in older adults with a history of falling. Gerontology & Geriatrics Research. 2016;2(3):1018.

10. Wu G, Keyes L, Callas P, Ren X, Bookchin B. Comparison of telecommunication, community, and home-based Tai Chi exercise programs on compliance and effectiveness in elders at risk for falls. Arch Phys Med Rehabil. 2010;91(6):849-56. doi: 10.1016/j.apmr.2010.01.024. PubMed PMID: 20510973.

11. Yerlikaya T, Öniz A, Özgùren M. The effect of an interactive tele rehabilitation program on balance in older individuals. Neurological Sciences and Neurophysiology. 2021;38(3):180-6.
